# Feasibility of positron range correction in 82-Rubidium cardiac PET/CT

**DOI:** 10.1186/s40658-022-00480-0

**Published:** 2022-07-30

**Authors:** Malte Jensen, Simon Bentsen, Andreas Clemmensen, Jacob Kildevang Jensen, Johanne Madsen, Jonas Rossing, Anna Laier, Philip Hasbak, Andreas Kjaer, Rasmus Sejersten Ripa

**Affiliations:** 1 Department of Clinical Physiology, Nuclear Medicine and PET and Cluster for Molecular Imaging, Copenhagen University Hospital – Rigshospitalet and Department of Biomedical Sciences, University of Copenhagen, Blegdamsvej 9, 2100 Copenhagen, Denmark; 2 Department of Clinical Physiology, Nuclear Medicine and PET, Copenhagen University Hospital – Rigshospitalet, Copenhagen, Denmark

**Keywords:** PET, MPI, Molecular imaging, Image reconstruction

## Abstract

**Background:**

Myocardial perfusion imaging (MPI) using positron emission tomography (PET) tracers is an essential tool in investigating diseases and treatment responses in cardiology. ^82^Rubidium (^82^Rb)-PET imaging is advantageous for MPI due to its short half-life, but cannot be used for small animal research due to the long positron range. We aimed to correct for this, enabling MPI with ^82^Rb-PET in rats.

**Methods:**

The effect of positron range correction (PRC) on ^82^Rb-PET was examined using two phantoms and in vivo on rats. A NEMA NU-4-inspired phantom was used for image quality evaluation (%standard deviation (%SD), spillover ratio (SOR) and recovery coefficient (RC)). A cardiac phantom was used for assessing spatial resolution. Two rats underwent rest ^82^Rb-PET to optimize number of iterations, type of PRC and respiratory gating.

**Results:**

NEMA NU-4 metrics (no PRC vs PRC): %SD 0.087 versus 0.103; SOR (air) 0.022 versus 0.002, SOR (water) 0.059 versus 0.019; RC (3 mm) 0.219 versus 0.584, RC (4 mm) 0.300 versus 0.874, RC (5 mm) 0.357 versus 1.197. Cardiac phantom full width at half maximum (FWHM) and full width at tenth maximum (FWTM) (no PRC vs. PRC): FWTM 6.73 mm versus 3.26 mm (true: 3 mm), FWTM 9.27 mm versus 7.01 mm. The in vivo scans with respiratory gating had a homogeneous myocardium clearly distinguishable from the blood pool.

**Conclusion:**

PRC improved the spatial resolution for the phantoms and in vivo at the expense of slightly more noise. Combined with respiratory gating, the spatial resolution achieved using PRC should allow for quantitative MPI in small animals.

## Introduction

Single-photon emission computed tomography (SPECT) and positron emission tomography (PET) are essential tools in diagnostics and characterization of various aspects of cardiovascular disease. Myocardial perfusion imaging (MPI) utilizes different radioisotopes, e.g., ^82^Rubidium (^82^Rb) and [^13^N]ammonia (^13^NH_3_) for PET and [^99m^Tc]Technetium-sestamibi (^99m^Tc-sestamibi) for SPECT, to enable the estimation of myocardial perfusion and myocardial blood flow (MBF) [1, 2]. ^82^Rb is used extensively in the clinical workout of patients with known or suspected coronary artery disease. An advantage of using ^82^Rb for MPI is the short half-life (76 s) that ensures low radiation exposure to the patients and it is produced using an on-site generator, circumventing the need for a cyclotron. ^82^Rb is a better predictor of cardiac outcome than ^99m^Tc-sestamibi, the scan time is shorter and it allows for dynamic images [3, 4]. [^18^F]Fluorine-Flurpiridaz is a potential tracer for MPI, but is not FDA approved and is currently only validated against ^99m^Tc-sestamibi [5]. The generator used for ^82^Rb is expensive, but a clinical ^82^Rb generator can be repurposed for preclinical research at little extra cost after ended clinical duty thereby making research in small animals with ^82^Rb economically feasible. ^13^NH_3_ in comparison would require the preclinical PET/CT scanner to be in close proximity to the cyclotron, a space which is usually reserved for the clinical scanners.

^82^Rb emits high-energy β^+^ decay, leading to a large distance from decay to annihilation. The distance from decay to annihilation is the positron range (PR), and a long PR lowers the spatial resolution on the reconstructed images. ^82^Rb has a mean PR of 7.5 mm compared to 1.7 mm of ^13^ N-ammonia [6].

When examining the human heart, the spatial resolution does not hinder the analysis of MPI. However, when examining smaller objects, e.g., the rodent heart, the PR of ^82^Rb lowers the spatial resolution to an extent where the myocardium and blood pool cannot be distinguished sufficiently, making analysis of MPI in rodent hearts unreliable [7].

Attempts to use ^82^Rb in rats undergoing myocardial infarction show that the images indeed have a low spatial resolution due to the long PR hindering a detailed analysis of MPI [7, 8], but despite this limitation ^82^Rb-uptake still correlate with the ejection fraction and infarct size [8]. If the long PR could be corrected, the spatial resolution would improve, allowing for more detailed analysis of MPI using ^82^Rb in small animals, which would be of value to cardiovascular research. Previous research has, e.g., shown that some cardiovascular drugs may alter the myocardial uptake of ^82^Rb in rats [9]. With a rat model of ^82^Rb, many more aspects of clinical significance could be investigated. However, positron range correction (PRC) on small animal cardiac ^82^Rb-PET imaging has not previously been published.

The distance from decay to annihilation is determined by the energy of the β^+^ decay and the density of the tissue. Therefore, the densities of the surrounding tissue are important in PRC. Models assuming homogeneous densities are fast and effective, but do not take into account the border zone between tissue [10–12]. The heart is located between tissues of very different densities (lung, muscle and bones), which need to be applied to the model in order to correct for the PR.

A simple model for multiple tissue densities is a segmentation from the computed tomography (CT) scan, which has previously been published; tissue-dependent (TD) PRC [13]: TD corrects separately for PR in each tissue segmented. The same group has also published a more advanced model using segmentation of the CT; tissue-dependent spatially variant PRC (TDSV): TDSV corrects for PR in each tissue segmented and the border zone between tissues [6, 11, 13, 14].

The aim of this study was to determine whether PRC using the TD or TDSV models applied to ^82^Rb-PET improved the spatial resolution in phantoms and in vivo. We hypothesize that PRC can increase the spatial resolution, allowing for the discrimination between blood pool and myocardium, enabling evaluation of MPI in small animals.

## Materials and methods

### Study design

The study consisted of both phantom and in vivo evaluation of PRC in ^82^Rb-PET/CT. Image quality evaluation was performed in a NEMA NU-4-inspired phantom and spatial resolution evaluation was performed in a cardiac phantom. Both phantoms were scanned with ^82^Rb-PET/CT using a preclinical PET/CT scanner (Siemens Inveon, Knoxville, TN, USA). For in vivo evaluation of PRC, a rat with and without infarction underwent ^82^Rb-PET/CT.

### NEMA NU-4 and cardiac phantom

**Fig. 1:**
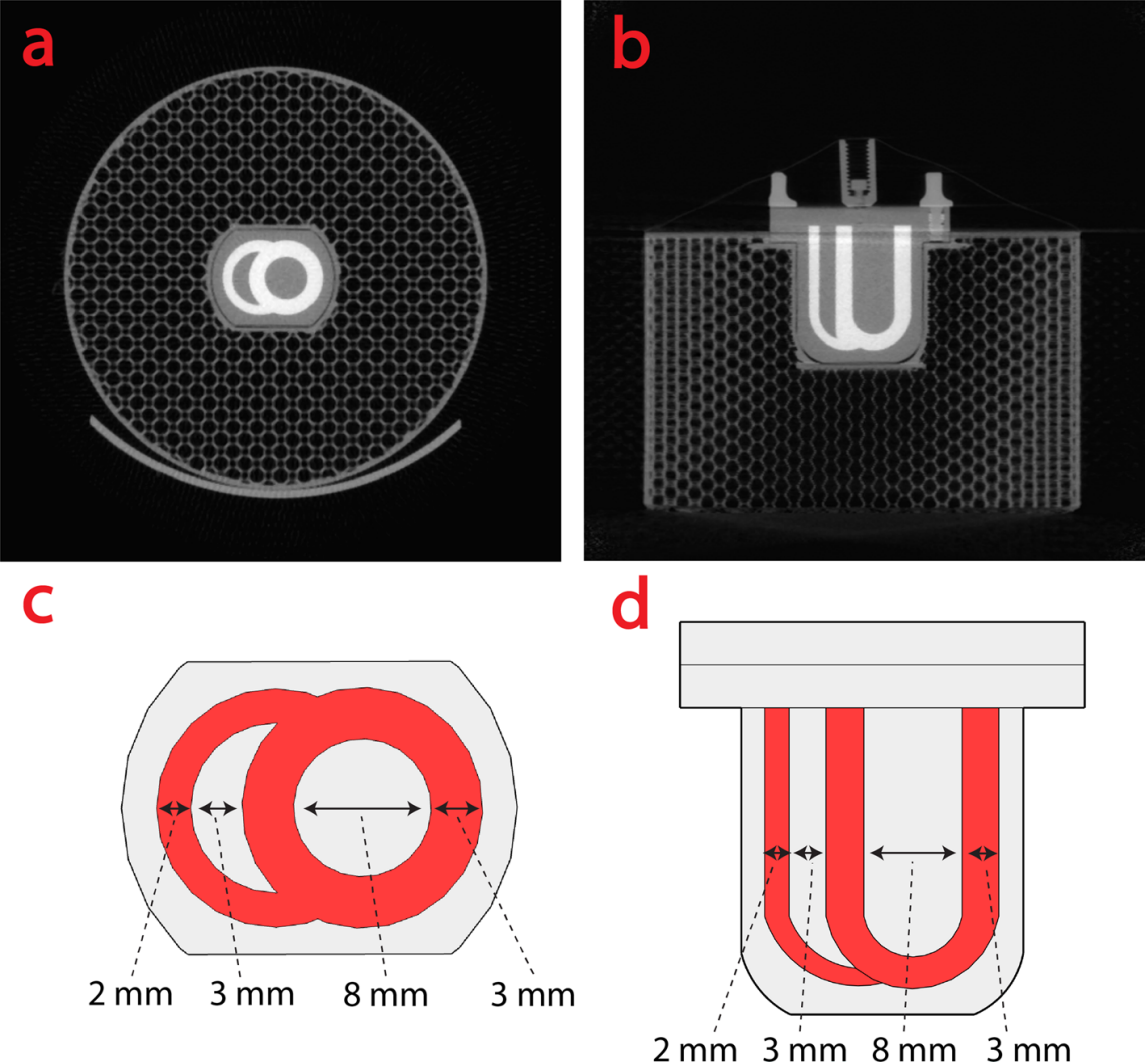
CT image of the cardiac phantom in **a** axial b coronal view. **c** and **d** are diagrams of the cardiac phantom in the same views as **a** and (**b**), respectively. CT = computed tomography

The NEMA NU-4-inspired phantom consists of three regions: uniform region, cold region and rod region [15]. The cardiac phantom consisted of a small two-compartment chamber inserted into a five cm cylinder (Fig. 1). The two-compartment chamber mimics the right and left ventricles, and the cylinder mimics the lung tissue. The cylinder was 3D printed (Prusa i3, Praha, Czech) to an average density of 0.4 g/cm^3^.

### Experimental animals

Outbred male Sprague Dawley rats were used for the animal study. The rats were 6 weeks of age when arriving at our facility. The rats were acclimatized 7 days before entering the study. The animals were housed in IVC Greenline Double Decker cage (Tecniplast Group, Italy). The cages were kept at 21 ± 2 °C with a 12:12 h dark:light cycle. The animals had access to water and standard rodent chow ad libitum.

The rats were anaesthetized using gas, 4% sevoflurane. The rats were intubated and ventilated To ensure proper ventilation (UNO micor-ventilator-O_3_, the Netherlands) throughout the surgery. During surgery, the rats were placed on a heated surface and pain treated with subcutaneously Buprenorphine 0.05 mL/kg. Prior to surgery, the chest of was shaved and iodine was used for sterilizing the skin.

An incision was then made in the skin and a thoracotomy was performed. The Insertion was placed at either the third or fourth intercostal space depending on size of the rat. The pericardium was identified and opened. Upon inspection of the heart, the left anterior descending artery (LAD) was identified and ligated 5 mm caudal of its origin with a 5–0 polypropylene suture. After suturering, the myocardium was observed for ischemia. If no discoloration of the myocardium was observed, another suture was made until ischemia was confirmed by discoloration. The thoracotomy was closed with suture type vicryl 4–0, in the muscle layer and skin. The first hours after surgery the rats were put in new cages with a heating lamp. The rats were pain treated four times per day the first 24 h and three times per day the next 72 h.

### PET acquisition

The phantoms and rats underwent PET/CT scan using the Siemens Inveon PET/CT scanner. The phantom was injected with ~ 50 MBq ^82^Rb and underwent a ten-minute PET acquisition. After the PET acquisition a CT scan was performed for segmentation and attenuation. The rats were anesthetized prior to PET/CT scan with Sevoflurane 3–4% and a 24G intravenous catheter (Vasofix Safety, Braun, Denmark) was placed in the tail vein. The rats were first CT scanned for attenuation correction. Then ~ 60 MBq ^82^Rb was administered and a dynamic 10-min PET scan was performed. The rats were monitored with ECG and a respiratory sensor during the PET/CT.

### Image reconstruction

The images of the two phantoms were histogrammed as a single static image with only one time frame. In the animal studies, the first 30 s of PET imaging was discarded, in order to have the ^82^Rb cleared from the blood. The dynamic images we histogrammed into bins of 1 × 6, 6 × 2, 2 × 5, 1 × 10, 1 × 20, 3 × 30, 1 × 452 s. Input curves were obtained in Corridor4DM version 2017 (Invia LLC, USA) using the Lortie method. The CT images were reconstructed using the Feldkamp cone beam algorithm to a size of 512 × 512 × 496 pixels. The open-source toolbox NiftyRec [16] allows for the implementation of PRC into the 3D Ordered Subset Expectation Maximization (3D-OSEM) algorithm, and the final PET images were reconstructed with either no PRC, TD PRC or TDSV PRC with 16 subsets for a target size of 128 × 128 × 159 pixels in the modified NiftyRec toolbox. The images were scatter-, attenuation- and prompt-gamma corrected. CT and PET images were anatomically registered to each other for all reconstructions.

### Respiratory gating

The in vivo rat studies was divided into 4 bins using respiratory gating. Each frame was reconstructed separately and subsequently summed together into a single static image. Scatter correction could not be applied to the gated images without losing too much information.

### TD and TDSV PRC

To produce the filter kernels (aPSF) for the PRC, the equation from Cal-Gonzáles et al. [6, 11, 13, 14] was used

$$aPSF\left( r \right) = C\left[ {\left( {a \cdot r + 1} \right)\left( {1 - \frac{r}{{r_{0} }}} \right)^{n} - \frac{\smallint }{{r^{n} }}} \right] \cdot \frac{1}{{r^{2} }}$$

where a, n, e and r0 are fitting parameters, and r is the Euclidean distance in millimeters. To scale the filter to different tissue densities, an equivalent distance was used

$$r_{eq} = r \cdot \rho_{j}$$

where ${\rho }_{j}$ is the density of tissue $j$. In the TDSV model, a straight line was drawn from the center to all neighboring voxels, and the mean of all the voxels penetrated out to a given voxel was used as ${\rho }_{j}$.

Results without PRC was named "no PRC." TD was named with the number of segmentation levels, such as "TD 2." TDSV was named with the size of the kernel, such as "TDSV 41." Water and air were segmented for the NEMA NU-4-inspired phantom and the cardiac phantom, and lung, water and bone for the in vivo studies.

### Data analysis

PET data were analyzed using Python, version 3.7 (Beaverton, OR, USA). In the NEMA NU-4-inspired phantom a region of interest (ROI) was drawn in each of the three compartments. The noise in the uniform region, the spillover ratio (SOR) in the cold region and the recovery coefficient (RC) in the rod region were evaluated from 1 to 100 iterations [15]. In the cardiac phantom, two lines were drawn. The first line represents a four-chamber view, showing the right and left ventricle. The second line represents a two-chamber view showing only the left ventricle. From each plane, a line profile was plotted showing the MBq/mL. To estimate the thickness of the ventricular walls, full width at half maximum at (FWHM) and full width at tenth maximum at (FWTM) were calculated.

To evaluate the in vivo rat ^82^Rb-PET the software Corridor4DM version 2017 (Invia LLC, USA) was used. The PET images were automatically oriented into short, vertical and long axis. A Butterworth filter of order 10 and a cutoff of 0.2 was applied.

## Results

### NEMA NU-4-inspired phantom

**Fig. 2:**
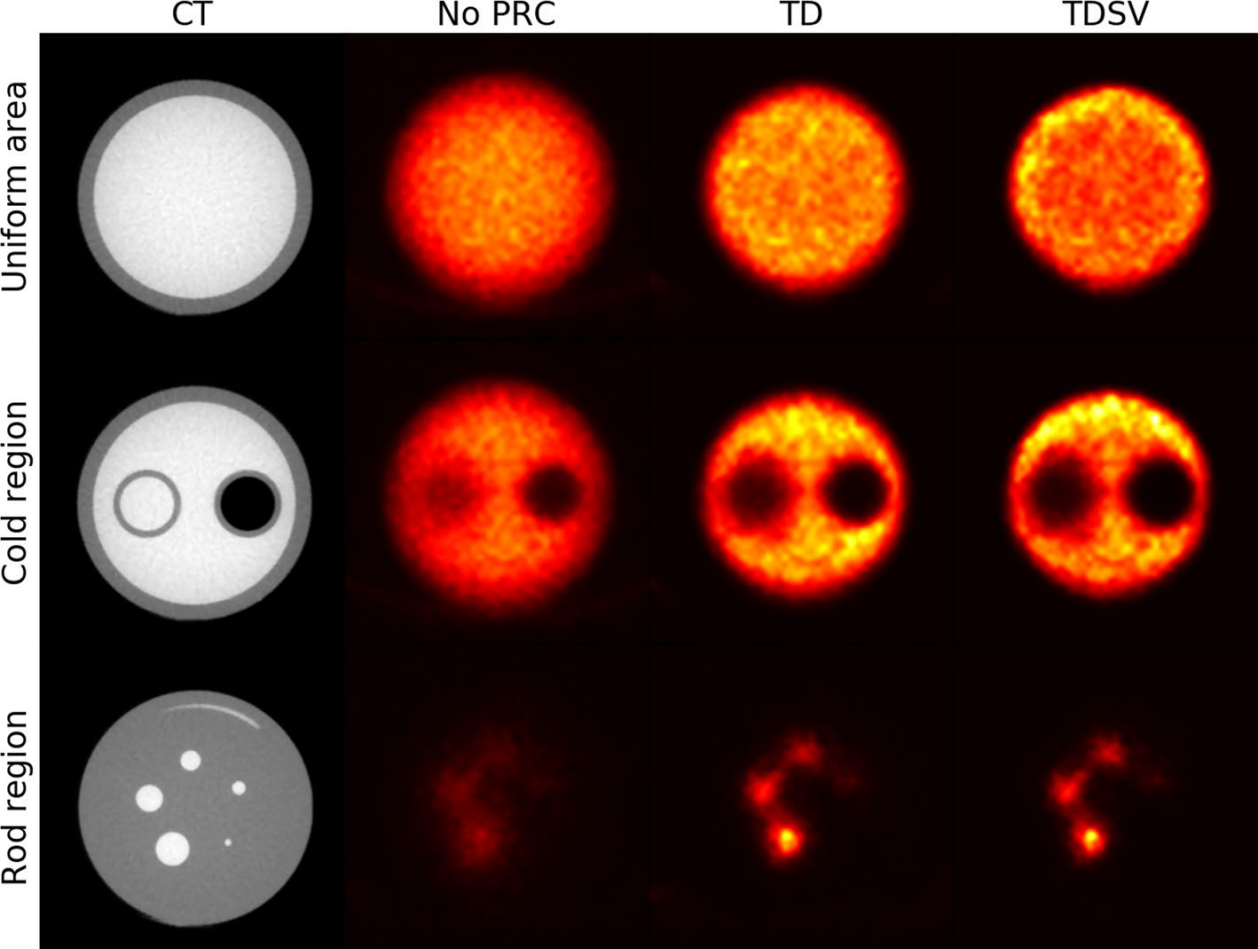
Axial view of the NEMA NU-4 phantom for the uniform, cold and rod region, shown for 40 iterations with no PRC, TD 2 and TDSV 41. All images are shown with the same intensity window. PRC = positron range correction; TD = tissue-dependent; TDSV = tissue-dependent spatially variant

An overview of the three different regions in the NEMA NU-4-inspired phantom with and without PRC is shown in Fig. 2. In the uniform area, the border between air and the phantom was more blurred with no PRC compared to the two PRC reconstructions. TDSV had a slight halo effect around the edges, also known as a Gibbs artifact [17]. In the left cylinder in the cold region (water), a great amount of spillover was present with no PRC compared to both PRC images. The spillover was more similar for all methods in the right air-filled cylinder, but the rod was still better delineated in the PRC images. In the rod region, the PRC reconstructions had more visible rods for 3–5 mm compared to the reconstruction without PRC, where the rods were more blurred.

#### Percent SD

**Fig. 3:**
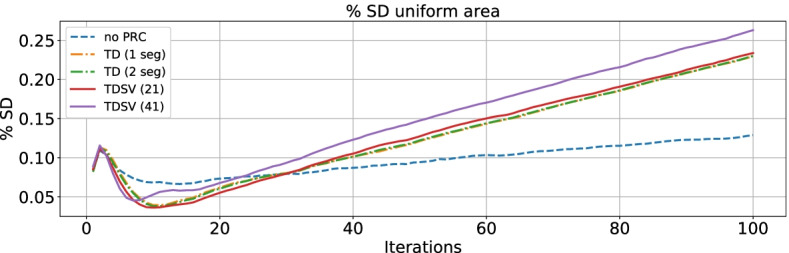
Percent standard deviation shown for 1–100 iterations with no PRC, TD 1 and 2, and TDSV 21 and 41. Abbreviations as in Fig. 2

The results are shown in Fig. 3. Overall, the no PRC method had a lower %SD until about 25 iterations, where the PRC curve superseded the no PRC curve. For all methods, the %SD kept increasing, but the PRC curves had a greater slope than no PRC, where TD 1 and 2 and TDSV 21 were very similar, while TDSV 41 had a slightly greater slope and also a higher offset than the other PRC methods.

#### Spillover ratio

**Fig. 4:**
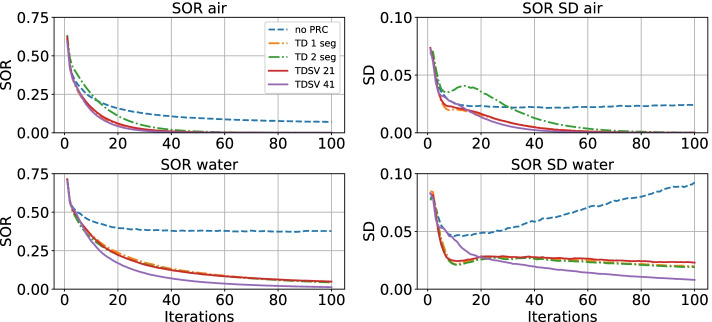
SOR shown for 1–100 iterations with no PRC, TD 1 and 2, and TDSV 21 and 41. SOR = spillover ratio, other abbreviations as in Fig. 2

The results are presented in Fig. 4 and Table 1. With no PRC, the SOR in the air-filled cylinder converged to about 0.1, while all PRC methods converged close to 0 after about 40 iterations. TD 2 converged slightly slower than the other PRC methods. The standard deviation (SD) of SOR in air converged to about 0.025 for no PRC and to 0 for all PRC methods after about 60 iterations.

**Table 1:** NEMA NU-4 metrics at 40 iterations

	No PRC	TD 1	TD 2	TDSV 21	TDSV 41
% SD	0.087	0.103	0.103	0.108	0.125
SOR air	0.022	0.005	0.012	0.004	0.002
SOR water	0.059	0.027	0.026	0.028	0.019
RC 1 mm	0.108	0.032	0.031	0.043	0.014
RC 2 mm	0.114	0.114	0.112	0.151	0.103
RC 3 mm	0.219	0.550	0.539	0.581	0.584
RC 4 mm	0.300	0.748	0.751	0.778	0.874
RC 5 mm	0.357	1.207	1.205	1.197	1.374

In the water-filled cylinder, no PRC converged to about 0.4 after 30 iterations, while the SOR for all PRC methods kept decreasing. TD 1 and 2, and TDSV 21 reached about 0.05 after 100 iterations while TDSV 41 reached around 0. The SD of SOR in water increased with more iterations for no PRC, while it kept decreasing for all the PRC methods. It decreased faster for TDSV 41 compared to the other PRC methods.

#### Recovery coefficient

**Fig. 5:**
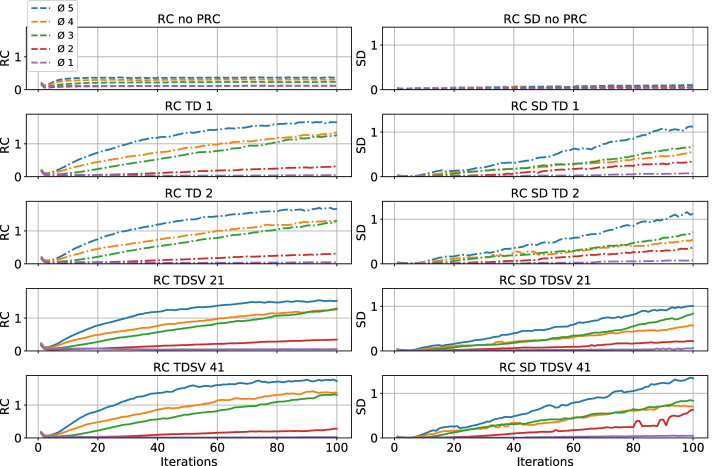
RC shown for 1–100 iterations with no PRC, TD 1 and 2, and TDSV 21 and 41. RC = recovery coefficient; SD = standard deviation, other abbreviations as in Fig. 2

The results are shown in Fig. 5 and Table 1. For no PRC, the RC converged after 10 iterations and was between 0.1 and 0.5. PRC mainly had an effect on the RC for the 3–5 mm rods, which all exceeded 1. The RC graphs for TD 1 and 2, and TDSV 21 were essentially identical, while the RC for TDSV 41 increased faster for the 3–5 mm rods. The SDs for no PRC were smaller than for the PRC methods.

#### Post-filtering

The effect of post-filtering with a Butterworth filter (order 10, cutoff 0.2) is shown in Figs. 9, 10, 11 and 12 and in Table 3. Filtering reduced the noise for %SD and RC, as well as preventing the RCs from rising much higher than 1, while the SOR is mostly unchanged. The Butterworth filter reduced the noise more for the TD methods than for the TDSV.

### Cardiac phantom

#### No PRC

**Fig. 6:**
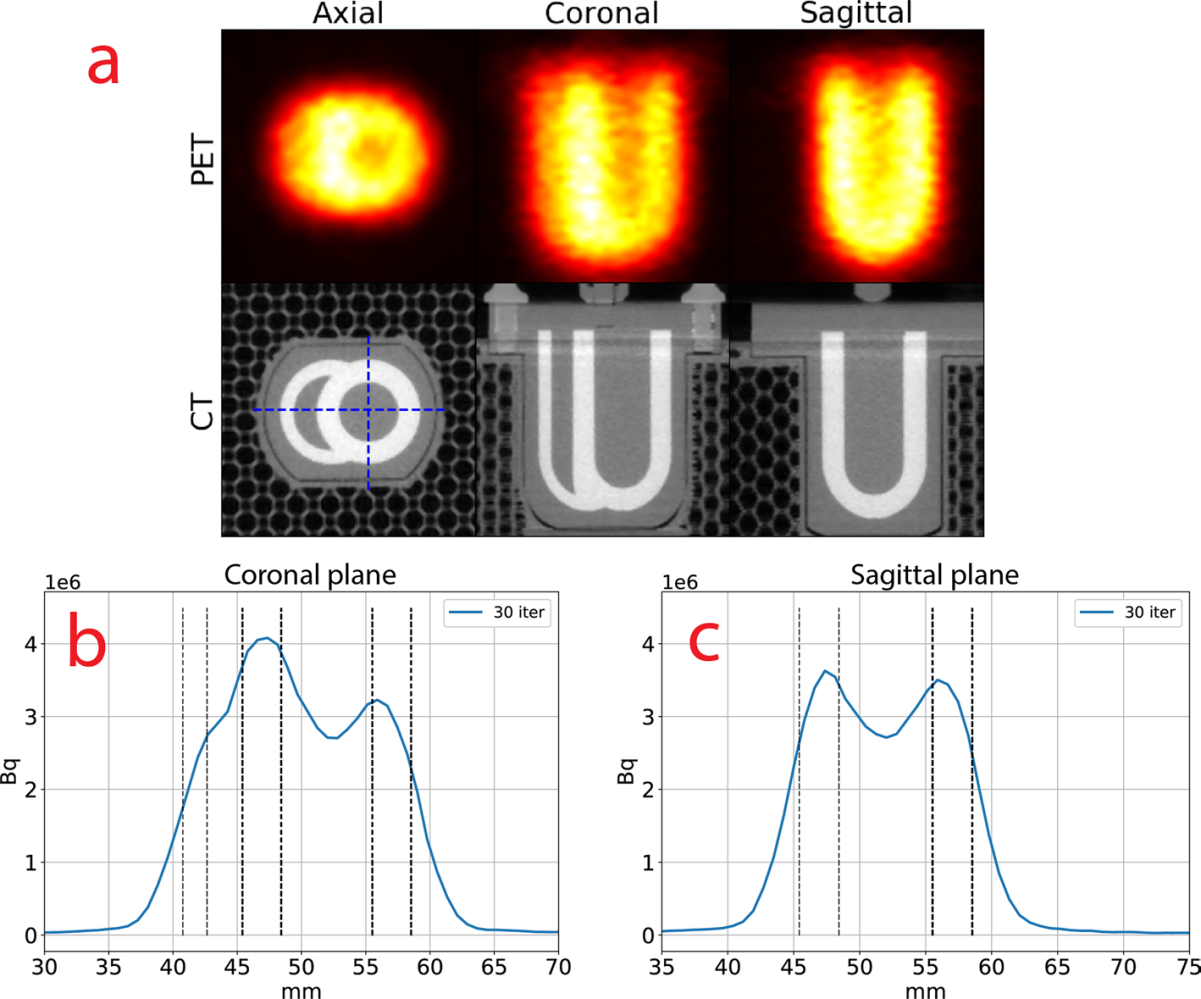
**a** PET image with no PRC and CT of the cardiac phantom, shown in the axial, coronal and sagittal plane for 30 iterations. The image was interpolated with B-splines. The blue crosshairs indicate where the coronal and sagittal line profiles were shown. Line profiles for the **b** Coronal **c** Sagittal planes. The black lines show the placement of the right ventricular wall (leftmost) and the left ventricular walls (two to the right). Bq = Becquerel; iter = iterations; PET = positron emission tomography; CT = computed tomography, other abbreviations as in Fig. 2

Figure 6 shows the PET/CT for the cardiac phantom with no PRC in 3 planes (axial, coronal and sagittal) and the line profiles of the phantom. The lumen of left and right ventricle could not be clearly discerned from the ventricular wall due to the amount of spillover on neither the PET image (Fig. 14) nor the line profiles (Fig. 6B, C). More than 30 iterations did not improve the image quality (data not shown).

#### TD 2

**Fig. 7:**
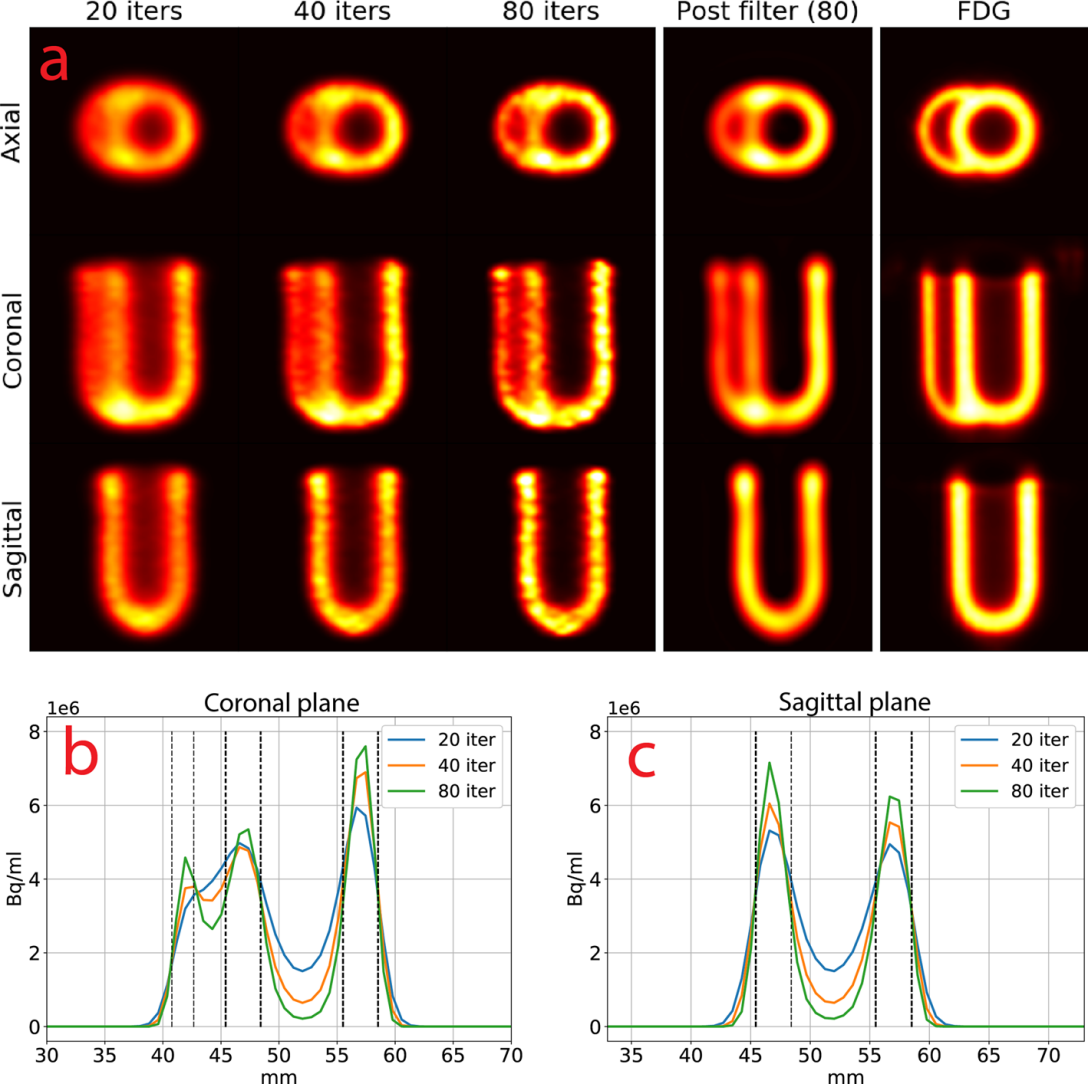
**a** Cardiac phantom with TDSV 41 for 20, 40 and 80 iterations, 80 iterations post-filtered with a Butterworth filter and an FDG PET scan. The images were interpolated with B- splines and shown for the same intensity window. Line profiles for the **b** coronal and **c** sagittal planes. The black lines show the placement of the right ventricular wall (leftmost) and the left ventricular walls (two to the right). Abbreviations as in Figs. 2, 3, 4, 5, 6 and 7

The results of TD 2 PRC are shown in Fig. 13 for 20, 40, 80 iterations with their line profiles, 80 iterations filtered with a Butterworth filter and an ^18^Fluorodeoxyglucose (FDG) scan of the phantom for comparison. Figure 13B and 7C show the line profiles. Only TD 2 is shown, as TD 1 gave identical images (Table 2).

**Table 2:** FWHM and FWTM of the left ventricular wall at 40 iterations. The true thickness was 3 mm

	No PRC	TD 1	TD 2	TDSV 21	TDSV 41
FWHM	6.73	3.44	3.47	3.74	3.26
FWTM	9.27	7.54	7.57	7.87	7.01

Compared to no PRC (Fig. 6), the right ventricular wall and lumen were clearly distinguishable. The spillover into the lumen of the left ventricle was almost eliminated at 80 iterations (Fig. 13B, C). The right ventricular wall and lumen could be seen after 80 iterations, but were not clearly delineated compared to the FDG scan. The difference in peak activity of the septum and lateral ventricular wall increased with more iterations (Fig. 13B), while it stayed similar in the anterior and inferior left ventricular wall (Fig. 13C).

More iterations also resulted in a more speckled PET image (Fig. 13A), which could be moothed by post-filtering the image with a Butterworth filter. Compared with the FDG scan, the FWHM and FWTM of the left ventricular wall (Table 2) improved substantially compared to no PRC.

#### TDSV 41

The results of TDSV 41 (Fig. 7) were similar to those of TD 2, with a few differences. At 40 and 80 iterations, the lumen of the left ventricle had less spillover than TD 2 (Fig. 13A, B). However, hot spots in the junction between the left and right ventricular wall appeared, both in the axial plane and at the apex in the coronal plane.

The lumen of the right ventricle was slightly more visible than for TD 2 (Fig. 7B), but the left ventricular lateral wall and the septum diverged more in peak activity. The activity of the anterior and inferior left ventricular walls also diverged slightly (Fig. 7C) compared to Fig. 13C.

The FWHM and FWTM of the left ventricular wall (Table 2) improved slightly compared to TD 1, 2 and TDSV 21.

### In vivo studies

The in vivo rat results show two aspects of PRC: The effect of iterations and the effect of respiratory gating with the different PRC methods.

#### Number of iterations

In Fig. 14, a PET image with no PRC and with TD 1 for 20, 40 and 80 iterations can be seen. With no PRC the blood pool and wall of the left ventricle could not be clearly discerned due to spillover. For TD 1, 20 iterations yielded a homogenous myocardium, but with spillover into the blood pool. With 40 iterations, the myocardium was slightly less homogeneous, but the blood pool was better delineated. At 80 iterations the blood pool was even better well delineated, but the myocardium developed hot spots. Since the absence of noise and artifacts was more important than higher spatial resolution, 40 iterations was chosen as the best trade-off for the remainder of the experiments.

#### PRC types and respiratory gating

**Fig. 8:**
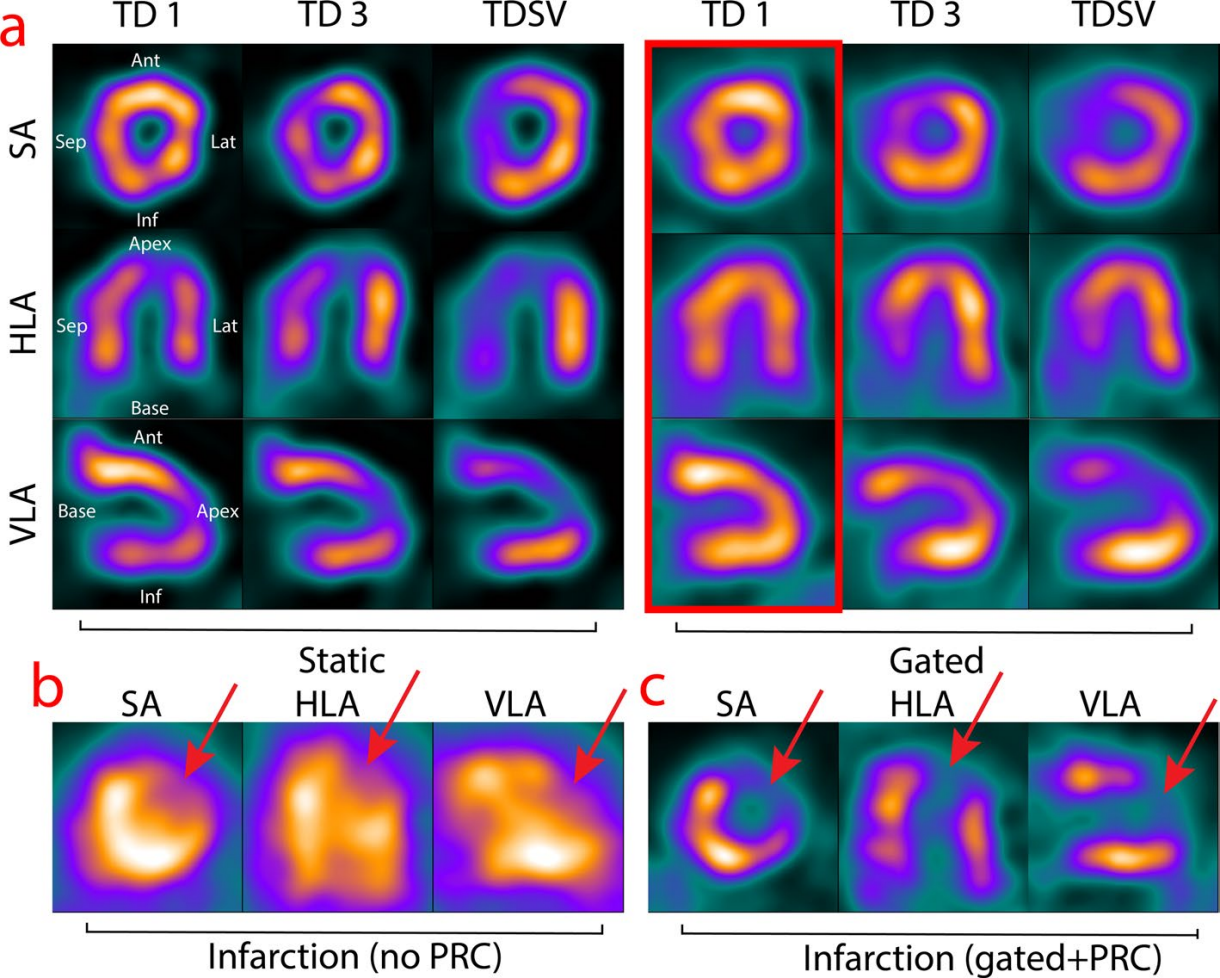
**a** Reconstruction of in vivo rat heart with TD 1, TD 3 and TDSV 41 for 40 iterations in the SA, HLA and VLA planes. The left group was static reconstructions and the right group was respiratory gated with 4 bins. The gated images were not scatter corrected. The red frame indicates the most optimal method. **b** and **c** shows an in vivo rat heart with an infarction reconstructed with no PRC and the chosen method in (**a**), respectively. The red arrows indicate the infarction, as opposed to the natural thinning of the apex. Abbreviations as in Figs. 2, 3, 4, 5, 6, 7, 8 and 9

**Fig. 9:**
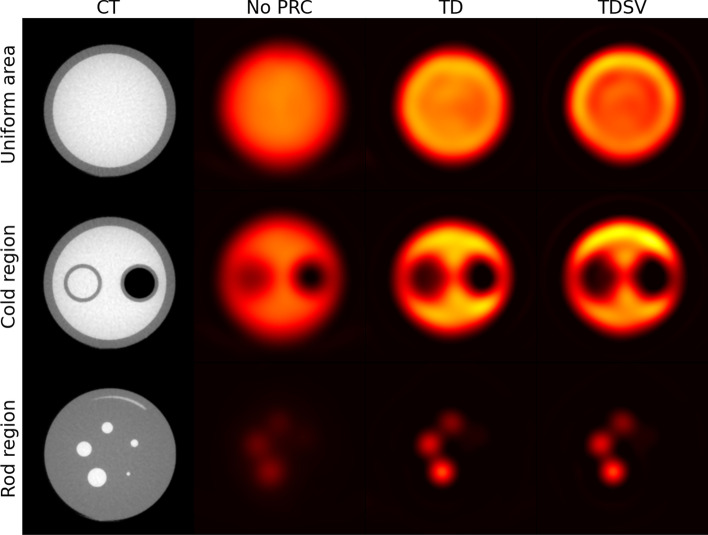
As in Fig. 2, but with a Butterworth filter with order 10 and cutoff 0.2

**Fig. 10:**
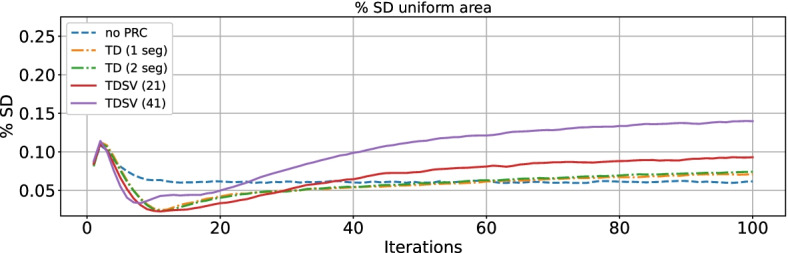
As in Fig. 3, but with a Butterworth filter with order 10 and cutoff 0.2

**Fig. 11:**
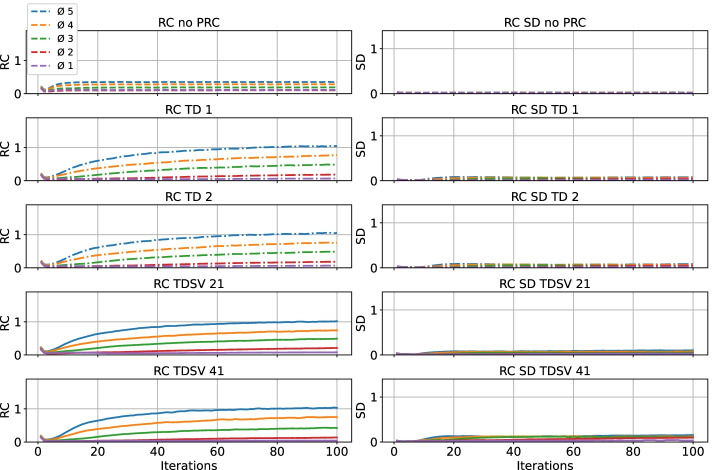
As in Fig. 4, but with a Butterworth filter with order 10 and cutoff 0.2

**Fig. 12:**
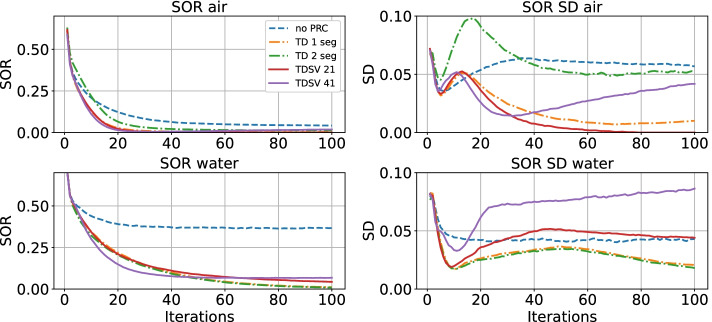
As in Fig. 5, but with a Butterworth filter with order 10 and cutoff 0.2

**Fig. 13:**
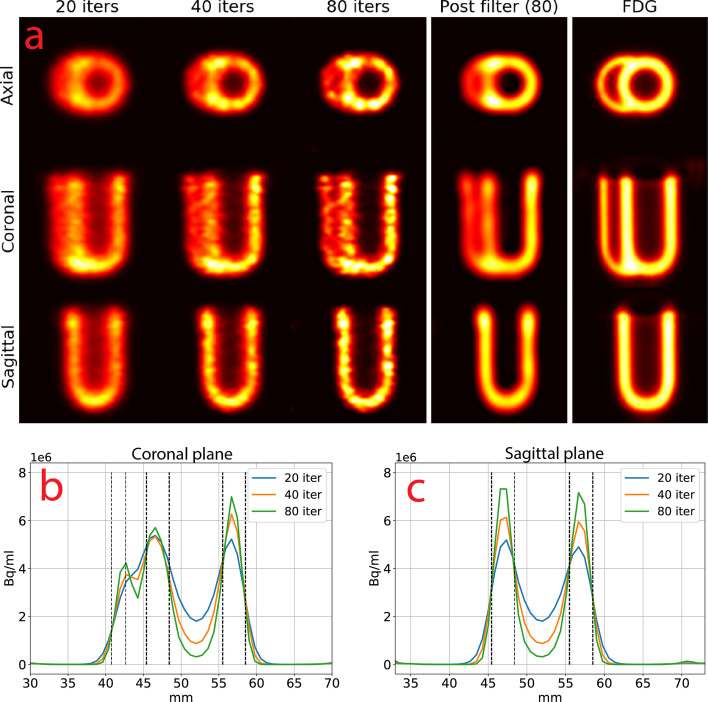
**a** Cardiac phantom with TD 2 for 20, 40 and 80 iterations, 80 iterations post-filtered with a Butterworth filter and an FDG PET scan. The images were interpolated with B-splines and shown for the same intensity window. Line profiles for the **b** coronal and **c** sagittal planes. The black lines show the placement of the right ventricular wall (leftmost) and the left ventricular walls (two to the right). FDG = fluoro-deoxyglucose, other abbreviations as in Figs. 2, 3, 4, 5 and 6

**Fig. 14:**
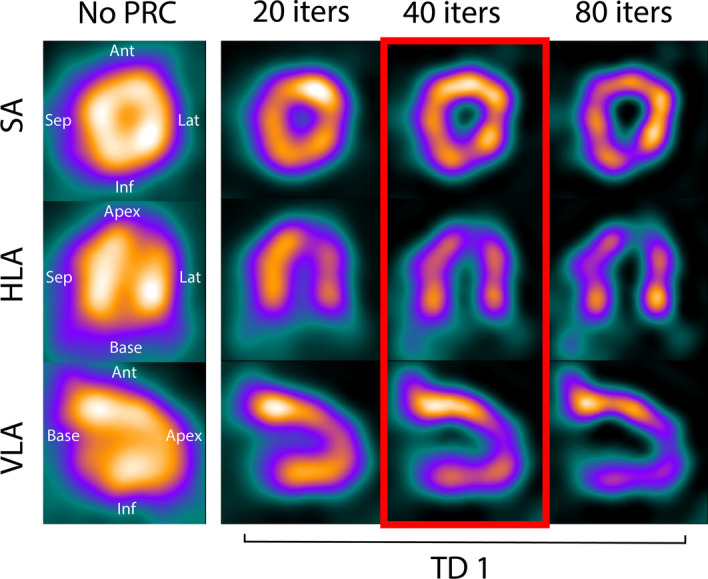
Reconstruction of a in vivo rat heart with TD 1 and no gating for 20, 40 and 80 iterations in the SA, HLA and VLA planes. The leftmost column shows no PRC. The red frame indicates the optimal number of iterations. SA = short axis; HLA = horizontal long axis; VLA = vertical long axis; ant = anterior; sep = septal; lat = lateral; inf = inferior, other abbreviations as in Figs. 2, 3, 4, 5, 6 and 7

The results of TD 1, TD 3 and TDSV 41 with and without respiratory gating are shown in Fig. 8A.

For the static images (Fig. 8A), TD 3 and TDSV 41 led the lateral and inferior walls (facing the lungs) to increase in activity and slightly shorten the extent of the inferior wall, where TD 1 conversely had more activity in the anterior wall.

Using 4 bins of respiratory gating and 40 iterations (Fig. 8A) yielded a homogenous myocardium for TD 1 while the inferior wall developed a hot spot for TD 3 and TDSV 41. More background scatter around the heart and in the blood pool were present due to the images not being scatter corrected. Since TD 1 with gating and 40 iterations was the most homogenous, this setup was used to reconstruct a rat with an infarction (Fig. 8C). The infarction could clearly be distinguished in the anteroapical part of the left ventricle with PRC applied, but it was difficult without (Fig. 8B).

#### Dynamic images

**Fig. 15:**
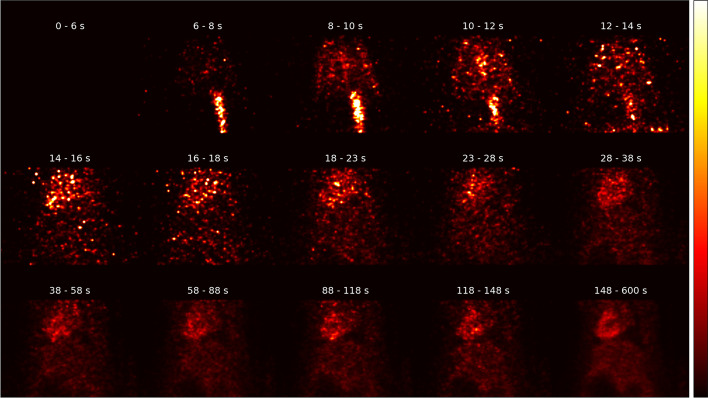
Gallery of the rat with no infarction, depicting each time frame in the coronal plane. Time bins in seconds are written in white on top of each image. In the bottom of each image is the vena cava and in the top the myocardium can be seen. All images are shown in the same intensity window

In Fig. 15, a gallery of the total rat without infarction can be seen for each time frame in the coronal view, where TD 1 PRC was applied. In the first ~ 15 s, the bolus of ^82^Rb can be seen in the vena cava, before it started to accumulate in the myocardium.

## Discussion

In this study, we present the first results using PRC on cardiac ^82^Rb-PET imaging in small animals. PRC increased the spatial resolution in all the experiments, but a trade-off between iterations and noise/artifacts needed to be made to obtain a sufficiently homogenous myocardium. In the in vivo studies, respiratory gating was essential for obtaining good quality images of the rat heart with PRC.

### NEMA NU-4-inspired phantom

The PRC methods improved the SOR and RC compared to no PRC, which is also visually evident from Fig. 2. This was at the expense of increased %SD and SD for the RC. The NEMA NU-4-inspired phantom was not optimal for showing the effect of different tissue densities, but still showed that TDSV 41 had a slight benefit over TD and TDSV 21 for SOR and RC, but at the expense of introducing more noise. From the RCs in the rod region, it appeared that the lower boundary for the PRC to work was around 2–3 mm. These results are in accordance with earlier research applying PRC to PET image reconstruction, where the spatial resolution and boundary delineation would increase, while the noise in the image would also increase, especially at boundaries between low and high activity [12, 18]. To the best of our knowledge, no research has demonstrated the effect of PRC for ^82^Rb in in a NEMA NU-4 phantom, but it has been demonstrated for ^89^zirconium [19], ^124^iodine [13] and ^68^gallium (^68^Ga) [11]. These studies also found an increase in %SD and RC, with a decrease in SOR.

### Cardiac phantom

PRC improved the delineation between the lumen and the myocardium of the left ventricle, while the right ventricle could not be clearly recovered. TD 1 and TD 2 produced almost identical images, while TDSV 41 improved the spatial resolution and clearance of the lumen slightly over the other methods, which was in line with our results for SOR and RC in the NEMA NU-4-inspired phantom. Higher resolution was, however, at the expense of producing hot spots, especially in the junctions of the phantom where the diameter was slightly greater and was most likely related to the RCs increasing faster for TDSV 41. The RCs indicated that 2–3 mm was the lower boundary for PRC being effective, which could explain why the right ventricle was not clearly reconstructed, having a wall thickness of 2 mm. Earlier research showed a resolution of ~ 1.5 mm for ^68^ Ga [11] with PRC, which have about half the PR of ^82^Rb. This fits well with the ~ 3 mm resolution we found for ^82^Rb in our experiments.

Since homogeneity of the myocardium is very important for clinical application of MPI, the cardiac phantom indicated that the TD 1 method would yield the best trade-off between spatial resolution and noise. Others have found TDSV to work better for tumors with ^68^ Ga [14], but the activity gradient toward the lungs was also small.

### In vivo studies

The distinction between the blood pool and myocardium was much clearer when applying PRC, but prone to hot spots. The anterior wall tended to develop hot spots with more iterations, which was alleviated with respiratory gating. Hence, this was most likely due to the movement of the heart, where the most static parts would appear to have a higher activity, while the activity of the more moving parts would be “thinned out.” As seen with the phantoms, this difference could be amplified by high iterations, leading to hot spots. Respiratory gating was hence a very important component of using PRC in vivo. ECG gating did not lead to qualitative improvement in the images (results not shown).

Both TD 3 and TDSV 41 had a tendency to introduce Gibbs artifacts [13] in the inferior and lateral wall of the heart, which were at the interface between the myocardium and the lung tissue. The activity gradient was big here, leading to a more difficult PRC task [20, 21]. The cross talk between the lungs and the myocardium is complex, and spillover in both directions occurs. Hence, spatially variant methods have only previously been shown to work in this case for simulations and with human-scale hearts, not with in vivo rat hearts. Despite spatially variant methods being successful for non-moving targets such as tumors [14], our results indicate that TD 1 produced more robust and reliable images for MPI, although the spatial resolution was higher for the spatially variant methods. Given that most of a rat consists of water-like tissue, it was not too surprising that TD 1 was still a good model. Since only two rats were included in this study, our in vivo results should be regarded as a proof of concept.

Figure 15 depicts a gallery of each time frame for the rat with no infarction in the coronal view. The bolus of ^82^Rb could be followed in the vena cava the first ~ 15 s, before it started to accumulate in the myocardium. This further indicates the potential to perform preclinical MPI in rats when PRC is applied.

In our experiments, the gated images could not be scatter corrected without losing too much information in the images. Better scatter correction from, e.g., Monte Carlo simulations [22] could likely improve the images further in future research, maybe leading to optimization of TDSV for MPI. In a clinical setting or with larger animals, the scatter correction will be of greater importance and should be used if PRC is needed.

## Conclusion

We demonstrated that the application of PRC in ^82^Rb MPI could increase the spatial resolution to a degree, where rodent models of myocardial disease could become feasible, in particular when applying respiratory gating on static images and without gating in the dynamic images.

## Data Availability

Not applicable.

## Appendix

See Figs. 9, 10, 11, 12, 13, 14, 15 and Table 3.

**Table 3:** NEMA NU metrics with Butterworth filter applied at 40 iterations

	No PRC	TD 1	TD 2	TDSV 21	TDSV 41
% SD	0.060	0.054	0.055	0.066	0.100
SOR air	0.063	0.016	0.057	0.007	0.018
SOR water	0.042	0.034	0.032	0.050	0.075
RC 1 mm	0.112	0.047	0.046	0.053	0.037
RC 2 mm	0.103	0.097	0.093	0.118	0.071
RC 3 mm	0.185	0.324	0.320	0.339	0.299
RC 4 mm	0.285	0.548	0.549	0.559	0.580
RC 5 mm	0.351	0.857	0.849	0.847	0.875

## Abbreviations

FWHM: Full width at half maximum
FWTM: Full width at tenth maximum
MBF: Myocardial blood flow
MPI: Myocardial perfusion imaging
PR: Positron range
PRC: Positron range correction
RC: Recovery coefficient
ROI: Region of interest
SOR: Spillover ratio
SPECT: Single-photon emission computed tomography
TD: Tissue-dependent
TDSV: Tissue-dependent spatially variant
